# Mesenchymal Stem Cells and Psoriasis: Systematic Review

**DOI:** 10.3390/ijms232315080

**Published:** 2022-12-01

**Authors:** Federico Diotallevi, Mariangela Di Vincenzo, Emanuela Martina, Giulia Radi, Vincenzo Lariccia, Annamaria Offidani, Monia Orciani, Anna Campanati

**Affiliations:** 1 Department of Clinical and Molecular Sciences, Dermatological Clinic, Università Politecnica delle Marche, 60126 Ancona, Italy; 2 Histology, Department of Clinical and Molecular Sciences, Università Politecnica delle Marche, 60126 Ancona, Italy; 3 Pharmacology, Department of Biomedical Sciences and Public Health, Università Politecnica delle Marche, 60126 Ancona, Italy

**Keywords:** mesenchymal stem cells, psoriasis, microenvironment, biologics, small molecules, systemic treatments

## Abstract

Mesenchymal Stem Cells (MSCs) are multipotent non-hematopoietic stromal cells found in different body tissues such as bone marrow, adipose tissue, periosteum, Wharton’s jelly, umbilical cord, blood, placenta, amniotic fluid, and skin. The biological behavior of MSCs depends mainly on their interaction with the microenvironment in which they are found, whose quality deeply influences the regenerative and immunomodulatory properties of these cells. Several studies confirm the interaction between MSCs and inflammatory microenvironment in the pathogenesis of psoriasis, designating MSCs as an important factor driving psoriasis development. This review aims to describe the most recent evidence on how the inflammatory microenvironment that characterizes psoriasis influences the homeostasis of MSCs and how they can be used to treat the disease.

## 1. Introduction

Psoriasis is considered a chronic, inflammatory, and immune-mediated systemic disease with involvement of both skin and other organs, including at least cardiovascular and articular systems and gastrointestinal district, with various degrees of involvement variable from patient to patient.

Psoriasis affects 0.51–11.43% of the adult population [1], and it is associated with a psychological burden that can lead to mental disorders, including depression and anxiety [2].

The introduction of molecular target treatments has been linked to the great improvement of patient’s quality of life and morbidity, and their safety profile has contributed to making these treatments the gold standard of treatment for psoriasis patients [3,4,5,6].

The phenomenon of the undertreatment and non-treatment of psoriasis is still diffuse worldwide, including Europe and the USA, and even worse in those developing countries where psoriasis patients with the moderate-to-severe disease have poor or not all access to these new expensive treatments [7,8]. A recent global survey named “Clear about Psoriasis” stated that 57% of moderate-to-severe psoriasis patients currently under treatment do not achieve clear/almost clear status [9], confirming earlier findings about most psoriasis patients worldwide are inadequately treated.

Main involved stakeholders in treating psoriasis patients agree that this global gap derives from several circumstances, and they call into question economic reasons, diversified market accesses, different health policies, misalignment between patient and physician perspectives in terms of disease severity and therapeutic goals, and finally knowledge gaps in psoriasis physiopathology.

For all these reasons, the development of novel therapeutic strategies for the management of psoriasis patients is universally recognized, including the use of mesenchymal stem cell-based therapies (MSCs) [10].

Indeed, several studies claim the skin is a source of MSCs [11,12], and those obtained from healthy subjects (H-MSCs) have an inherent anti-inflammatory and immunomodulatory activity due to the blockade of T lymphocyte proliferation and differentiation obtained through different mechanisms, which include cell cycle arrest, secretion of soluble anti-inflammatory factors such as transforming growth factor B, hepatocyte growth factor, and prostaglandin E2. In addition, MSCs are also able to promote tolerance of antigen-presenting cells, reducing the phenomena of antigen processing and presentation to naive T lymphocytes [13].

In individuals with psoriasis, however, it appears that MSCs are negatively affected by the inflammatory microenvironment in which they are found: these cells, defined as PsO-MSCs, become dysfunctional and no longer have anti-inflammatory but pro-inflammatory activity, contributing to the pathogenesis of the disease through interaction with keratinocytes and immune cells [14,15].

The aim of this systematic review is to report the best evidence on MSC’s involvement in psoriasis, focusing on their interaction with the microenvironment an understanding of both how these cells may contribute to the development of the disease and the possibility that these cells may underlie new therapeutic strategies.

## 2. Methods

**Figure 1:**
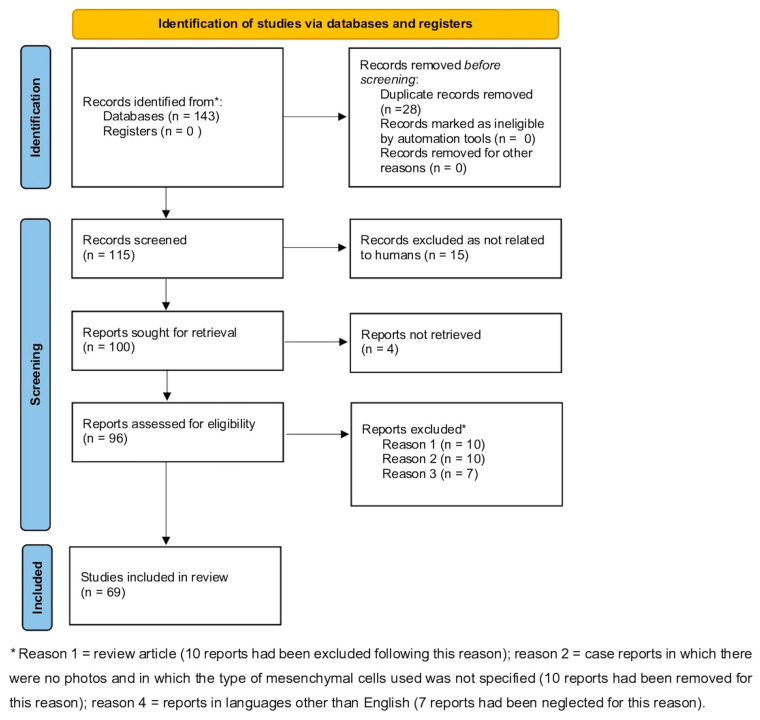
Preferred Reporting Items for Systematic Reviews and Meta-Analysis (PRISMA) on the interaction between MSCs and microenvironment in psoriasis [17].

This scoping review was based on the approach developed by Arksey and O’Malley [16] that includes five essential steps: identification of the research question; identification of appropriate studies; selection of studies; tracking of data; and collection, summarization, and reporting of results. The Preferred Reporting Items for Systematic Reviews and Meta-Analysis (PRISMA) extension for scoping review criteria was used to guide the conduction and reporting of the review [17] (Figure 1).

### 2.1. Identification of the Research Question

A brainstorming approach involving the entire research team was used to identify the research questions. The research group included five dermatologists with expertise in the research field of psoriasis and clinical management of psoriatic patients, two histologists, and one pharmacologist, all with experience in the research field of MSCs in inflammatory and immune-mediated skin diseases.

At the initial meeting, the group identified the research question and determined the research strategy. The research question was: “how can the interaction between MSCs and the microenvironment influence psoriasis development and therapies?”.

### 2.2. Study Selection Process

We performed a worldwide systematic review of studies reporting on MSCs and psoriasis, using 3 electronic medical databases–PubMed, EMBASE, and Web of Science, considering articles dated 1 January 2012 to 1 April 2022.

The search terms were selected to identify studies describing the relationship between MSCs and psoriasis.

The keywords used were “mesenchymal stem cell(s), or mesenchymal stromal cell(s) or mesenchymal stem/stromal cell(s) AND psoriasis”, “mesenchymal stem cell(s) or mesenchymal stromal cell(s) or mesenchymal stem/stromal cell(s) AND psoriasis AND microenvironment”, “mesenchymal stem cell(s), or mesenchymal stromal cell(s) or mesenchymal stem/stromal cell(s) AND psoriasis AND therapies”, “mesenchymal stem cell(s) or mesenchymal stromal cell(s) or mesenchymal stem/stromal cell(s) AND psoriasis AND biologics”, “mesenchymal stem cell(s) or mesenchymal stromal cell(s) or mesenchymal stem/stromal cell(s) AND psoriasis AND small molecules”.

All selected databases were searched from their respective inception. In addition, we searched by hand the reference lists of other relevant articles on MSCs and psoriasis.

In this first phase, 143 records were identified from selected databases. Records, after duplicates were removed, 115. Among selected records, none were marked as ineligible by automation tools. Relevant studies were then chosen. This process occurred in three phases. In the first phase, four researchers (A.C., F.D., E.M., G.R., M.O.) independently selected articles based on the title. Any disagreements were resolved by consulting a senior researcher (A.O.). In the second phase, abstracts were evaluated. Four members of the research team (A.C., M.O., M.D.V., V.L.) independently evaluated each abstract. The research group resolved all discrepancies through unanimous consent. Fifteen-five articles were excluded, and 100 were evaluated for full-text analysis. Among them, 4 manuscripts were not retrieved. Thus documents assessed for eligibility were 96.

The third phase consisted of a critical appraisal of the full text of the 96 selected papers.

To be included in our mini-review, manuscripts should have contained a complete characterization of the cell populations studied, according to the International Society of Cell Therapy [https://www.isctglobal.org/home, accessed on 29 October 2022] which indicated whether the function of MSC populations was really being analyzed. Moreover, studies had to be focused on the involvement of MSCs in psoriasis development, clinical course, and response to systemic therapies. All included studies had to be published in English, with abstracts available. No restrictions on study design were considered, and in vitro and in vivo pre-clinical trials, controlled clinical trials, case-control studies, cross-sectional studies, and case series were included. Articles were excluded from our review for three reasons only: reason 1 = review article (10 reports had been excluded following this reason), reason 2 = case reports in which there were no photos and in which the type of mesenchymal cells used was not specified (10 reports had been removed for this reason), reason 3 = reports in languages other than English (7 reports had been neglected for this reason). PRISMA is shown in Figure 1.

### 2.3. Data Extraction

A data extraction module was designed by A.C. before data extraction to accelerate the entire process. In order to answer the research question, the following information was extracted from the included articles: Author(s) name and publication date; study design; study population; sample size; measured outcomes; study results; and study recommendations. The flowchart of the PRISMA study is shown in Figure 1. Our search identified 115 records after removing duplicates. After a review of titles and abstracts, 15 citations were dropped (research not related to humans or reports not retrieved), and 4 documents were not retrieved. Thus 96 were evaluated for full-text eligibility. After review of the full text, 69 pre-clinical trial, controlled clinical trial, case-control study, cross-sectional studies, and case series were found to be eligible and included in this study.

## 3. Results and Discussion

The interaction between MSCs and the microenvironment plays a role in many pathogenetic and therapeutic aspects of psoriasis. The increase in knowledge on the interaction between MSCs and the psoriatic microenvironment represents a key requirement to understand the future positioning of regenerative medicine in this field.

### 3.1. Interaction between PsO-MSCs and Inflammatory Microenvironment in Psoriasis

The interaction between the microenvironment and MSCs is very complex and based on different mechanisms, such as secretion of soluble factors, crosstalk with other cell types, and release of vesicles or non-coding RNAs. It has been established that MSCs derived from the skin of psoriatic patients (PsO-MSCs) show an immunomodulatory profile that reflects the Th1-Th17/Th2 imbalance of psoriasis with upregulation of genes encoding Th1 and Th17 cytokines [14,15].

This genetic profile, together with the interaction with keratinocytes, fibroblasts, and with immunity cells, results in the maintenance of the inflammation characteristic of the disease [15].

#### 3.1.1. Relationships between PsO-MSCs and Psoriatic Keratinocytes (PsO-KCs)

**Figure 2:**
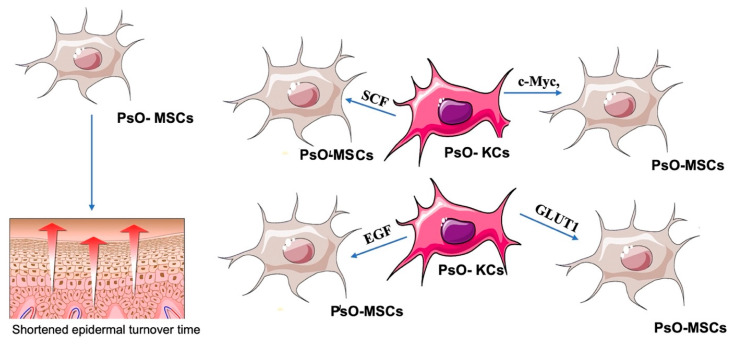
Cross-talking between PsO-MSCs and PsO-KCs: High levels of pro-inflammatory cytokines secreted by MSCs induce KCs proliferation, shortening the epidermal turnover time through secretion of several cytokines including Epidermal Growth Factor (EGF). PsO-KCs co-cultured with control MSCs upregulates the expression of c-Myc, GLUT1, SCF, and EGF, resulting in enhanced metabolism and increased proliferation of MSCs, establishing a vicious cycle.

Keratinocytes (KCs) play essential roles in both the initiation and maintenance phases of psoriasis since, being a part of the innate immune system, KCs are responsive to multiple triggers. There is mutual crosstalk between MSCs and KCs: on the one hand, high levels of pro-inflammatory cytokines secreted by MSCs induce KCs proliferation, probably via activating the PI3K/AKT signaling pathway [18] and through the secretion of cytokines such as Epidermal Growth Factor (EGF) [19] and inhibit their apoptosis due to the reduction in expression levels of caspase-3 [20,21]; it has also been observed that dermal PsO-MSCs contribute to the reduction in the cellular junction, with a consequent alteration in KC differentiation and shortened epidermal turnover time [22,23]. On the other hand, PsO-KCs co-cultured with control MSCs upregulates the expression of c-Myc, GLUT1, SCF, and EGF, resulting in enhanced metabolism and increased proliferation of MSCs, establishing a vicious cycle [24] (Figure 2).

#### 3.1.2. Effects of PsO-MSCs on the Vascularization of Psoriatic Lesions

**Figure 3:**
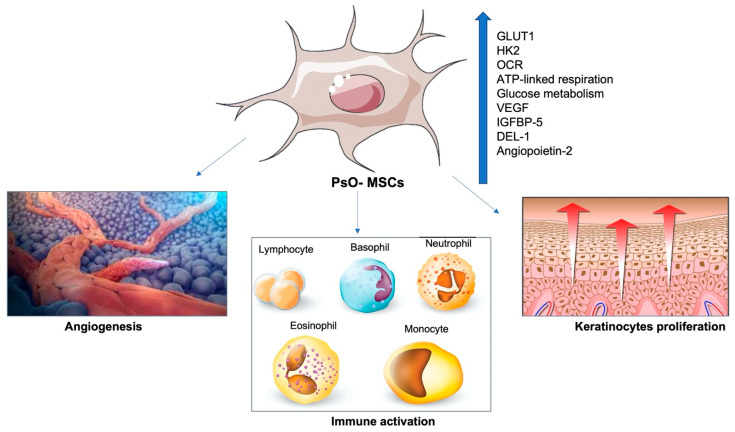
Effects of PsO-MSCs on the vascularization of psoriatic lesions: upregulation of GLUT1, HK2, OCR, ATP-linked respiration, glucose metabolism, VEGF, IGFBP-5, DEL-1 and angiopoietin-2 in dermal PsO-MSCs promotes immune activation, keratinocytes proliferation, and angiogenesis in inflamed skin.

An upregulation of GLUT1 and HK2 has also been found in dermal PsO-MSCs [25]. OCR (oxygen consumption rate), ATP-linked respiration, maximal respiration, and reserve capacity are significantly higher in PsO-MSCs than in Control-MSCs C/MSCs. Upregulation of glucose metabolism is thought to promote hyperproliferation, inflammation, and angiogenesis in psoriasis. Vascular hyperplasia is evident in psoriatic lesions. It was shown that the number of blood vessels in the psoriatic skin significantly increased compared to the control group [25]. Overexpression of pro-angiogenic mediators such as VEGF, IGFBP-5, DEL-1, and angiopoietin-2 in dermal PsO-MSCs has been consolidated [14,26,27] (Figure 3).

Also, co-cultures with endothelial cells (ECs) have demonstrated that PsO-MSCs influence the adhesion and migration of ECs by upregulating the expression of integrins αVβ3 and α5β1 [26,27]. However, the mechanisms remain unknown. The study of other pro-angiogenic molecules (PECAM1, FGD5, PTGS1, MCAM, VASH2, and STAB 1 has revealed a reduction in their expression in PsO-MSCs, suggesting that other pathways could be involved in angiogenesis and vascular dilatation [28,29].

#### 3.1.3. Relationships between PsO-MSCs and Immune Cells

**Figure 4:**
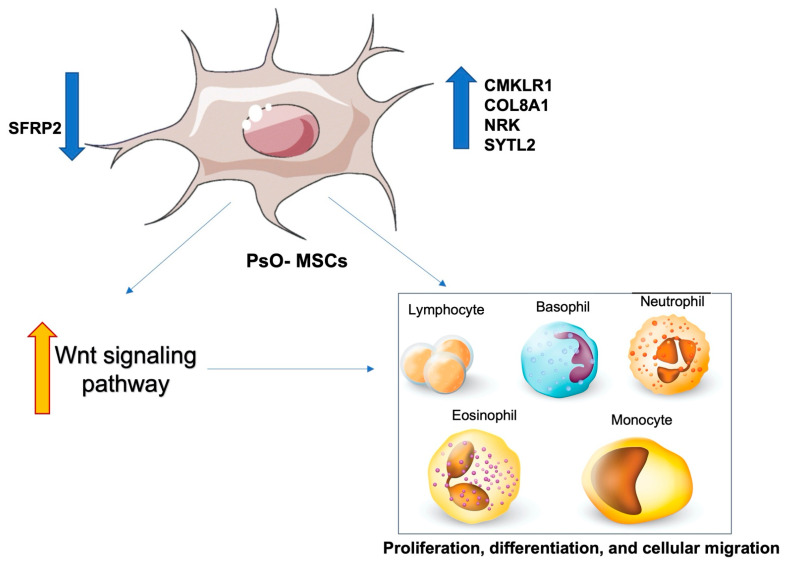
Relationships between PsO-MSCs and immune cells: overexpression of CMKLR1, COL8A1, NRK, and SYTL2 in PsO-MSCs promotes proliferation, differentiation, and migration of healthy peripheral blood mononuclear cells (PBMCs), including lymphocytes, basophils, neutrophils, eosinophils ad monocytes, through the activation of Wnt signaling pathway.

Neovascularization is a method used by PsO-MSCs to achieve inflammation through the enhanced migration of white blood cells to the skin site [30]. In fact, the expression of proteins involved in cellular migration (CMKLR1, COL8A1, NRK, and SYTL2) is upregulated in PsO-MSCs, affecting the migration of healthy peripheral blood mononuclear cells (PBMCs) [31,32]. Furthermore, PsO-MSCs have been proven to induce a downregulation of SFRP2 that enhances the activation of the Wnt signaling pathway that manages proliferation, differentiation, and cellular migration (Figure 4) [32].

In contrast, co-cultures between C-MSCs and PsO-MSCs have shown that C-MSCs exhibit a stronger inhibitory effect than PsO-MSCs on T cell proliferation, cytokines production, and apoptosis [33].

#### 3.1.4. PsO-MSCs and Micro-RNAs (miRNAs)

PsO-MSCs are also able to modulate the microenvironment through the non-coding micro-RNAs (miRNAs) [34,35].

In particular, the Circular RNA (circRNA) gene chr2:206992521|206994966 is mostly expressed by PsO-MSCs and affects the activity of T lymphocytes in local lesions by influencing their pro-inflammatory cytokine secretion (IL-6, IL-11, and hepatocyte growth factor) [36]. Another miRNA expressed by PsO-MSCs is MicroRNA-155 (MiR-155), which is involved in the pathogenesis of both inflammation and glucose metabolism in psoriasis and regulates the expression levels of various cytokines and enhances the glycolysis in psoriasis [37].

### 3.2. The Immunomodulatory Effect of “Healthy MSCs” on the Psoriasis Microenvironment

The interaction between healthy MSCs (H-MSCs) and the inflammatory microenvironment in psoriasis results in a global immunomodulatory effect. Indeed, it has been widely accepted that MSCs have immunosuppressive capabilities. Briefly, MSCs inhibit the proliferation of activated T cells, modulate the release of inflammatory cytokines and chemokines by dendritic cells and macrophages, suppress proliferation and immunoglobulin production of B cells, and inhibit cytotoxic activity of natural killer (NK) cells [38]. However, as mentioned above, the pro-inflammatory environment of psoriatic skin, in turn, affects the homeostasis of MSCs by reducing their anti-inflammatory effects [39]. Liu et al. cultured dermal PsO-MSCs in vitro with activated T cells and found that, compared with their normal counterpart, psoriatic patient-derived MSCs showed less ability to inhibit T cell proliferation [40]. In addition, Hou et al. found that, when stimulated by IFN-γ along with TNFα or IL-1β, MSCs showed reduced immunosuppressive capacity, with a significantly increased pro-inflammatory expression of miR-155 and inducible nitric oxide synthase (iNOS) [41]. In contrast, Campanati et al., demonstrated that in vitro H-MSCs can exert a strong paracrine effect by secreting soluble active factors that can restore the anti-inflammatory phenotypic profile of PsO-MSCs. Through the realization of indirect co-culture of H-MSCs with PsO-MSCs, researchers demonstrated that before co-culture, a proliferation of PsO-MSCs was significantly higher than H-MSCs and the levels of secreted cytokines confirmed the imbalance of Th1/Th17 versus the Th1 axis. After co-culture of H-MSCs with PsO-MSCs, healthy MSCs seem to exert a ‘positive’ influence on PsO-MSCs, driving the inflammatory phenotypical profile of PsO-MSCs towards a physiological pattern. Indeed, after the procedure, both the decrease in proliferation rate towards values closer to those observed in H-MSCs and the secretion of cytokines that mostly identified the inflammatory microenvironment that characterized psoriasis, such as interleukin (IL)-6, IL-12, IL-13, IL-17A, tumor necrosis factor (TNF)-a and granulocyte-macrophage colony-stimulating factor (G-CSF) were demonstrated [42]. From this evidence, strands of translational research developed to attempt to restore stem cell homeostasis in psoriatic patients either by using MSCs derived from healthy individuals or by using biological drugs [43,44,45].

#### 3.2.1. Effects of H-MSCs Administration in Psoriasis

Thus, the success of MSCs therapy in psoriasis has been demonstrated in both mouse and human models. In 2019, Kim et al. investigated the use of human embryonic stem cell-derived MSCs (hE-MSCs) on the imiquimod (IMQ)-induced skin psoriasis mouse model [46]. The researchers demonstrated that the clinical skin score was significantly reduced in the group with intradermal administration of E-MSCs compared with the IMQ control group. In histological analysis, they found that IMQ-induced epidermal thickness was significantly reduced by treatment with E-MSCs. In addition, they detected a marked reduction in Th1 cytokines (TNF-α, IFN-α, IFN-γ, and IL-27) and Th17 cytokines (IL-17A and IL-23) in the serum and skin of hE-MSCs-treated mice [46]. Similar results were achieved by Chen et al. in 2019, in which infusion of human umbilical cord-derived MSC (hUC-MSC) successfully produced improvement of psoriasis in mouse models via suppression of neutrophil function and then production of type I interferon (IFN-I) by plasmacytoid dendritic cells (pDCs) [47]. More recently, successful MSCs therapy on mouse models has been achieved through the topical application of Mesenchymal stem/stromal cell (MSC) exosomes preparations that have immunomodulatory properties and can attenuate inflammation by enhancing the secretion of anti-inflammatory cytokines, promoting Treg polarization and inhibiting complement activation. Thus, topically applied MSC exosomes in a mouse model of IMQ-psoriasis significantly reduced IL-17 and the C5b-9 terminal complement activation complex in mouse skin [48]. In addition, the stem cell secretome can be manipulated and made more suitable for therapeutic purposes for psoriasis. Indeed, the same research group demonstrated the efficacy of administering small extracellular vesicles derived from huc-MSCs transduced with a mirna with anti-inflammatory properties (miR-210) in mouse models of psoriasis [49].

Experiments on the use of H-MSCs in psoriasis have also been performed in humans. In 2016, Chen et al. described two cases of psoriasis Vulgaris successfully treated with infusion of umbilical cord-derived mesenchymal stem cells (UC-MSCs) with remission maintained for 5 years without relapse [50].

Also, in the same year, De Jesus reported the case of two patients multi failure to multiple therapies for psoriasis effectively treated with infusions of autologous MSCs derived from subcutaneous adipose tissue [51].

More recently, Wang et al. treated a 19-year-old man with a 5-year history of severe plaque psoriasis refractory to multiple topical and systemic therapies with an infusion of allogeneic human gingival MSCs. Complete regression was achieved after 5 infusions with no adverse reactions, and he experienced 3 years of disease-free status [52].

#### 3.2.2. Effects of Immunomodulatory Therapies on PsO-MSCs

Finally, the anti-inflammatory and immunomodulatory nature of MSCs in psoriatic patients would appear to be restored during biological therapies. Campanati et al., in fact, demonstrated a change in the expression of VEGF, iNOS, and Indoleamine 2,3-dioxygenase IDO as well as the restoration of pathological Th1-Th17/Th2 imbalance of MSCs during therapy with anti-TNF-alpha and apremilast [53,54].

## 4. Conclusions and Future Perspectives

Several previous studies have already demonstrated the involvement of MSCs in the pathogenesis of psoriasis. However, if psoriasis inflammatory pathways could induce MSCs dysfunction or primary MSCs dysregulation promotes psoriasis development, it remains to be determined. Moreover, it is still unclear whether the local dysfunction of MSC in psoriatic plaques is only a localized phenomenon or if it reflects a failure in MSC’s functioning in general.

Overall, the literature data indicate that MSCs obtained from psoriasis patients interact with inflammatory background becoming pro-inflammatory and feeding skin inflammation in turn.

PsO-MSCs are psoriasis committed: most part of the genes encoding for Th1 and Th17 cytokines (INF-γ, CXCL9, CXCL10, IL6, IL8, TNF-α, IL23A, CCL2, CCL20, CXCL2, CXCL5, IL17C, IL17F, IL17RA, IL21, CCR5, TLR2) is over-expressed in MSCs isolated from psoriasis, than healthy subjects. They may contribute to the overall pathogenetic process of psoriasis by promoting hyperplasia of epidermal keratinocytes, angiogenesis, activation, and infiltration of T lymphocytes, dendritic cells, neutrophils, and other types of leucocytes in the affected skin [14,15,54].

However, MSCs could also be considered as “Ianus Bifrons” cells, and naive unconditioned MSCs show immunomodulating effects against principal inflammation-aggravating pathways in psoriasis.

Classically, early studies showed that MSCs could exert an anti-inflammatory effect through inhibition of proliferation and differentiation of Th lymphocytes towards Th1-Th2-Th17 lineage and promotion of Treg expression. Moreover, MSCs are also able to condition the tolerogenic profile of antigen-presenting cells, reducing the following inflammatory cascade.

MSCs are able to exert these immunomodulatory effects through various mechanisms, including induction of cell cycle arrest [13,55], direct cell-to-cell contact [13,56], secretion of soluble mediators such as hepatocyte growth factor (HGF), transforming growth factor β1 (TGF-β1) and prostaglandin E2 (PGE2), and activation of a cytosolic enzyme such as IDO [56,57,58].

However, it is not clear whether the administration of MSCs may be able to reprogram the inflammatory axis in psoriatic patients and for how long this immunomodulatory effect can produce effective clinical improvement for patients because the only data available come from case reports.

Anyway, despite these uncertainties, MSCs have already been considered potential therapeutic targets for several immune-mediated and inflammatory skin diseases [10,45,59], and nowadays, some clinical trials on the use of MSCs for a great variety of autoimmune disorders, including psoriasis, are ongoing.

However, we are still very far from being able to think of a translational implication in the clinical field of the research in progress, as published studies show wide variation for the type of MSCs used and methods of administration, therefore making it impossible at present to define a clinical protocol.

Despite the promising rationale for the use of MSCs in psoriasis, it is mandatory to postulate that the in vivo regenerative and immunomodulatory potential effect of these cells may not be as effective as expected. In addition, potential side effects associated with regenerative therapy, including graft-versus-host disease, and localized cutaneous reactions, should also be considered.

Another problem with mesenchymal stem cell therapy could be the accumulation of cell mutations during proliferation in culture. Hyunjun Ahn’s group has proposed using “minimally manipulated umbilical cord MSCs” as a therapy for psoriasis to limit mutagenicity [60].

To overcome this problem, moreover, other researchers such as Zhang et al. have proposed the use of stem cell secretome instead of whole cell infusion. The researchers claim that the small extracellular vesicles (EVs) produced by MSCs could potentially be more effective and have fewer side effects than whole-cell therapy. Research in this area could evolve toward producing new mesenchymal stem cell secretome-based topical therapies that could potentially be effective [48,49].

The efficacy and safety profile of MSCs treatment varies according to the source of isolation and the method of administration. Isolation of MSCs from bone marrow requires a specialist team, whereas isolation from adipose tissue is simpler and potentially less burdened by side effects.

Another important topic to consider is the autologous or allogeneic nature of MSCs to be used: the immunomodulatory greater property of allogeneic compared to autologous MSCs seems to be an advantage that could increase the success rate of a treatment.

For this reason, cryopreservation of allogeneic cells could become an important issue to consider in the next years [61].

Recent studies have also established that the way of thawing is also important to avoid a failure of regenerative therapies, demonstrating that culturing these cells for 24 h after thawing allows for recovery of functional potency of allogenic MSCs and even an increased immunosuppressive capacity [62].

Assessment of the balance between risks and benefits is essential for MSC-based treatments to be considered a treatment for psoriasis in real-world settings [63,64].

They represent crucial items to be addressed in future studies aimed at determining the feasibility and efficacy of autologous or allogenic MSCs transplantation.

## Data Availability

Not applicable.

## References

[1] Michalek I.M., Loring B., John S.M. (2017). A systematic review of worldwide epidemiology of psoriasis. J. Eur. Acad. Derm. Venereol..

[2] Blackstone B., Patel R., Bewley A. (2022). Assessing and Improving Psychological Well-Being in Psoriasis: Considerations for the Clinician. Psoriasis.

[3] Armstrong A.W., Robertson A.D., Wu J., Schupp C., Lebwohl M.G. (2013). Undertreatment, treatment trends, and treatment dissatisfaction among patients with psoriasis and psoriatic arthritis in the United States: Findings from the National Psoriasis Foundation surveys, 2003–2011. JAMA Dermatol..

[4] Gisondi P., Cazzaniga S., Chimenti S., Maccarone M., Picardo M., Girolomoni G., Naldi L. (2015). Psocare Study Group. Latent tuberculosis infection in patients with chronic plaque psoriasis: Evidence from the Italian Psocare Registry. Br. J. Dermatol..

[5] Bardazzi F., Magnano M., Campanati A., Loconsole F., Carpentieri A., Potenza C., Bernardini N., Di Lernia V., Carrera C., Raone B. (2017). Biologic Therapies in HIV-infected Patients with Psoriasis: An Italian Experience. Acta Derm. Venereol..

[6] Campanati A., Moroncini G., Ganzetti G., Pozniak K.N., Goteri G., Giuliano A., Martina M., Liberati G., Ricotti F., Gabrielli A. (2013). Adalimumab modulates angiogenesis in psoriatic skin. Eur. J. Inflamm..

[7] Lebwohl M.G., Kavanaugh A., Armstrong A.W., Van Voorhees A.S. (2016). US perspectives in the management of psoriasis and psoriatic arthritis: Patient and physician results from the population-based multinational assessment of psoriasis and psoriatic arthritis (MAPP) survey. Am. J. Clin. Dermatol..

[8] World Health Organization (2016). World Health Organization Global Report on Psoriasis.

[9] Armstrong A., Jarvis S., Boehncke W.H., Rajagopalan M., Fernández-Peñas P., Romiti R., Bewley A., Vaid B., Huneault L., Fox T. (2018). Patient perceptions of clear/almost clear skin in moderate-to-severe plaque psoriasis: Results of the Clear About Psoriasis worldwide survey. J. Eur. Acad. Dermatol. Venereol..

[10] Paganelli A., Tarentini E., Benassi L., Kaleci S., Magnoni C. (2020). Mesenchymal stem cells for the treatment of psoriasis: A comprehensive review. Clin. Exp. Dermatol..

[11] Vaculik C., Schuster C., Bauer W., Iram N., Pfisterer K., Kramer G., Reinisch A., Strunk D., Elbe-Bürger A. (2012). Human dermis harbors distinct mesenchymal stromal cell subsets. J. Investig. Dermatol..

[12] Park J.R., Kim E., Yang J., Lee H., Hong S.H., Woo H.M., Park S.M., Na S., Yang S.R. (2015). Isolation of human dermis derived mesenchymal stem cells using explants culture method: Expansion and phenotypical characterization. Cell Tissue Bank..

[13] Shin T.H., Kim H.S., Choi S.W., Kang K.S. (2017). Mesenchymal Stem Cell Therapy for Inflammatory Skin Diseases: Clinical Potential and Mode of Action. Int. J. Mol. Sci..

[14] Orciani M., Campanati A., Salvolini E., Lucarini G., Di Benedetto G., Offidani A., Di Primio R. (2011). The mesenchymal stem cell profile in psoriasis. Br. J. Dermatol..

[15] Campanati A., Orciani M., Consales V., Lazzarini R., Ganzetti G., Di Benedetto G., Di Primio R., Offidani A. (2014). Characterization and profiling of immunomodulatory genes in resident mesenchymal stem cells reflect the Th1-Th17/Th2 imbalance of psoriasis. Arch. Dermatol. Res..

[16] Arksey H., O’Malley L. (2005). Scoping studies: Towards a methodological framework. Int. J. Soc. Res. Methodol..

[17] Page M.J., McKenzie J.E., Bossuyt P.M., Boutron I., Hoffmann T.C., Mulrow C.D., Shamseer L., Tetzlaff J.M., Akl E.A., Brennan S.E. (2021). The PRISMA 2020 statement: An updated guideline for reporting systematic reviews. BMJ.

[18] Liang N., Chang W., Peng A., Cao Y., Li J., Wang Y., Jiao J., Zhang K. (2022). Dermal Mesenchymal Stem Cells from Psoriatic Lesions Stimulate HaCaT Cell Proliferation, Differentiation, and Migration via Activating the PI3K/AKT Signaling Pathway. Dermatology.

[19] Liu R., Yang Y., Yan X., Zhang K. (2013). Abnormalities in cytokine secretion from mesenchymal stem cells in psoriatic skin lesions. Eur. J. Dermatol..

[20] Chang W., Liang N., Cao Y., Xing J., Li J., Li J., Zhao X., Li J., Niu X., Hou R. (2021). The effects of human dermal-derived mesenchymal stem cells on the keratinocyte proliferation and apoptosis in psoriasis. Exp. Dermatol..

[21] Liu R.F., Wang F., Wang Q., Zhao X.C., Zhang K.M. (2015). Research Note Mesenchymal stem cells from skin lesions of psoriasis patients promote proliferation and inhibit apoptosis of HaCaT cells. Genet. Mol. Res..

[22] Wang Y., Liang Y., Li J., Hou R., Li J., Liang N., Xing J., Jiao J., Chang W., Li X. (2020). Expression and functional regulation of gap junction protein connexin 43 in dermal mesenchymal stem cells from psoriasis patients. Acta Histochem..

[23] Li J., Xing J., Lu F., Chang W., Liang N., Li J., Wang Y., Li X., Zhao X., Hou R. (2020). Psoriatic Dermal-derived Mesenchymal Stem Cells Reduce Keratinocyte Junctions, and Increase Glycolysis. Acta Derm. Venereol..

[24] Cao Y., Liang N.N., Chang W.J., Li J.Q., Jiao J.J., Hou R.X., Li J., Zhang K.M. (2022). Role of psoriatic keratinocytes in the metabolic reprogramming of dermal mesenchymal stem cells. Int. J. Dermatol..

[25] Zhao X., Xing J., Li J., Hou R., Niu X., Liu R., Jiao J., Yang X., Li J., Liang J. (2021). Dysregulated Dermal Mesenchymal Stem Cell Proliferation and Differentiation Interfered by Glucose Metabolism in Psoriasis. Int. J. Stem Cells.

[26] Niu X., Han Q., Li X., Li J., Liu Y., Li Y., Wu Y. (2022). Del-1 in Psoriasis Induced the Expression of αvβ3 and α5β1 in Endothelial Cells. Curr. Mol. Med..

[27] Hou R., Yan H., Niu X., Chang W., An P., Wang C., Yang Y., Yan X., Li J., Liu R. (2014). Gene expression profile of dermal mesenchymal stem cells from patients with psoriasis. J. Eur. Acad. Dermatol. Venereol..

[28] Han Q., Niu X., Hou R., Li J., Liu Y., Li X., Li J., Li Y., Zhang K., Wu Y. (2021). Dermal mesenchymal stem cells promoted adhesion and migration of endothelial cells by integrin in psoriasis. Cell Biol. Int..

[29] Niu X., Chang W., Liu R., Hou R., Li J., Wang C., Li X., Zhang K. (2016). Expression of pro-angiogenic genes in mesenchymal stem cells derived from dermis of patients with psoriasis. Int. J. Dermatol..

[30] Zhou L., Wang J., Liang J., Hou H., Li J., Li J., Cao Y., Li J., Zhang K. (2021). Psoriatic mesenchymal stem cells stimulate the angiogenesis of human umbilical vein endothelial cells in vitro. Microvasc. Res..

[31] Castro-Manrreza M.E., Bonifaz L., Castro-Escamilla O., Monroy-García A., Cortés-Morales A., Hernández-Estévez E., Hernández-Cristino J., Mayani H., Montesinos J.J. (2019). Mesenchymal Stromal Cells from the Epidermis and Dermis of Psoriasis Patients: Morphology, Immunophenotype, Differentiation Patterns, and Regulation of T Cell Proliferation. Stem Cells Int..

[32] Niu X., Li J., Zhao X., Wang Q., Wang G., Hou R., Li X., An P., Yin G., Zhang K. (2019). Dermal mesenchymal stem cells: A resource of migration-associated function in psoriasis?. Stem Cell Res. Ther..

[33] Zhao X., Jiao J., Li X., Hou R., Li J., Niu X., Liu R., Yang X., Li J., Liang J. (2021). Immunomodulatory effect of psoriasis-derived dermal mesenchymal stem cells on TH1/TH17 cells. Eur. J. Dermatol..

[34] Li J., Hou R., Niu X., Liu R., Wang Q., Wang C., Li X., Hao Z., Yin G., Zhang K. (2016). Comparison of microarray and RNA-Seq analysis of mRNA expression in dermal mesenchymal stem cells. Biotechnol. Lett..

[35] Wang Q., Chang W., Yang X., Cheng Y., Zhao X., Zhou L., Li J., Li J., Zhang K. (2019). Levels of miR-31 and its target genes in dermal mesenchymal cells of patients with psoriasis. Int. J. Dermatol..

[36] Liu R., Chang W., Li J., Cheng Y., Dang E., Yang X., Wang Q., Wang G., Li X., Zhang K. (2019). Mesenchymal stem cells in psoriatic lesions affect the skin microenvironment through circular RNA. Exp. Dermatol..

[37] Liu Y., Zhao X., Li J., Zhou L., Chang W., Li J., Hou R., Li J., Yin G., Li X. (2022). MiR-155 inhibits TP53INP1 expression leading to enhanced glycolysis of psoriatic mesenchymal stem cells. J. Dermatol. Sci..

[38] Kim K.H., Blasco-Morente G., Cuende N., Arias-Santiago S. (2017). Mesenchymal stromal cells: Properties and role in management of cutaneous diseases. J. Eur. Acad. Derm. Venereol..

[39] Hou R., Li J., Niu X., Liu R., Chang W., Zhao X., Wang Q., Li X., Yin G., Zhang K. (2017). Stem cells in psoriasis. J. Dermatol. Sci..

[40] Liu R., Wang Y., Zhao X., Yang Y., Zhang K. (2014). Lymphocyte inhibition is compromised in mesenchymal stem cells from psoriatic skin. Eur. J. Dermatol..

[41] Xu C., Ren G., Cao G., Chen Q., Shou P., Zheng C., Du L., Han X., Jiang M., Yang Q. (2013). miR-155 regulates immune modulatory properties of mesenchymal stem cells by targeting TAK1-binding protein 2. J. Biol. Chem..

[42] Campanati A., Orciani M., Sorgentoni G., Consales V., Mattioli Belmonte M., Di Primio R., Offidani A. (2018). Indirect co-cultures of healthy mesenchymal stem cells restore the physiological phenotypical profile of psoriatic mesenchymal stem cells. Clin. Exp. Immunol..

[43] Owczarczyk-Saczonek A., Krajewska-Włodarczyk M., Kruszewska A., Placek W., Maksymowicz W., Wojtkiewicz J. (2017). Stem Cells as Potential Candidates for Psoriasis Cell-Replacement Therapy. Int. J. Mol. Sci..

[44] Vizoso F.J., Eiro N., Costa L., Esparza P., Landin M., Diaz-Rodriguez P., Schneider J., Perez-Fernandez R. (2019). Mesenchymal Stem Cells in Homeostasis and Systemic Diseases: Hypothesis, Evidences, and Therapeutic Opportunities. Int. J. Mol. Sci..

[45] Chen Y., Yu Q., Hu Y., Shi Y. (2019). Current Research and Use of Mesenchymal Stem Cells in the Therapy of Autoimmune Diseases. Curr. Stem Cell Res. Ther..

[46] Kim C.H., Lim C.Y., Lee J.H., Kim K.C., Ahn J.Y., Lee E.J. (2018). Human Embryonic Stem Cells-Derived Mesenchymal Stem Cells Reduce the Symptom of Psoriasis in Imiquimod-Induced Skin Model. Tissue Eng. Regen. Med..

[47] Chen M., Peng J., Xie Q., Xiao N., Su X., Mei H., Lu Y., Zhou J., Dai Y., Wang S. (2019). Mesenchymal Stem Cells Alleviate Moderate-to-Severe Psoriasis by Reducing the Production of Type I Interferon (IFN-I) by Plasmacytoid Dendritic Cells (pDCs). Stem Cells Int..

[48] Zhang B., Lai R.C., Sim W.K., Choo A.B.H., Lane E.B., Lim S.K. (2021). Topical Application of Mesenchymal Stem Cell Exosomes Alleviates the Imiquimod Induced Psoriasis-Like Inflammation. Int. J. Mol. Sci..

[49] Zhang W., Lin J., Shi P., Su D., Cheng X., Yi W., Yan J., Chen H., Cheng F. (2022). Small Extracellular Vesicles Derived from MSCs Have Immunomodulatory Effects to Enhance Delivery of ASO-210 for Psoriasis Treatment. Front. Cell Dev. Biol..

[50] Chen H., Niu J.W., Ning H.M., Pan X., Li X.B., Li Y., Wang D.H., Hu L.D., Sheng H.X., Xu M. (2016). Treatment of Psoriasis with Mesenchymal Stem Cells. Am. J. Med..

[51] De Jesus M.M., Santiago J.S., Trinidad C.V., See M.E., Semon K.R., Fernandez M.O., Chung F.S. (2016). Autologous Adipose-Derived Mesenchymal Stromal Cells for the Treatment of Psoriasis Vulgaris and Psoriatic Arthritis: A Case Report. Cell Transplant.

[52] Wang S.G., Hsu N.C., Wang S.M., Wang F.N. (2020). Successful Treatment of Plaque Psoriasis with Allogeneic Gingival Mesenchymal Stem Cells: A Case Study. Case Rep. Dermatol. Med..

[53] Campanati A., Caffarini M., Diotallevi F., Radi G., Lucarini G., Di Vincenzo M., Orciani M., Offidani A. (2021). The efficacy of in vivo administration of Apremilast on mesenchymal stem cells derived from psoriatic patients. Inflamm. Res..

[54] Campanati A., Orciani M., Lazzarini R., Ganzetti G., Consales V., Sorgentoni G., Di Primio R., Offidani A. (2017). TNF-α inhibitors reduce the pathological Th1 -Th17 /Th2 imbalance in cutaneous mesenchymal stem cells of psoriasis patients. Exp. Dermatol..

[55] Glennie S., Soeiro I., Dyson P.J., Lam E.W., Dazzi F. (2005). Bone marrow mesenchymal stem cells induce division arrest anergy of activated T Cells. Blood.

[56] English K., Ryan J.M., Tobin L., Murphy M.J., Barry F.P., Mahon B.P. (2009). Cell contact, prostaglandin E2 and transforming growth factor β1 play non-redundant roles in human mesenchymal stem cell induction of CD4+CD25high forkhead box p3+ regulatory T Cells. Clin. Exp. Immunol..

[57] Di Nicola M., Carlo-Stella C., Magni M., Milanesi M., Longoni P.D., Matteucci P., Grisanti S., Gianni A.M. (2002). Human bone marrow stromal cells suppress T-lymphocyte proliferation induced by cellular or nonspecific mitogenic stimuli. Blood.

[58] Aggarwal S., Pittenger M.F. (2005). Human mesenchymal stem cells modulate allogeneic immune cell responses. Blood.

[59] Orciani M., Campanati A., Caffarini M., Ganzetti G., Consales V., Lucarini G., Offidani A., Di Primio R. (2017). T helper (Th)1, Th17 and Th2 imbalance in mesenchymal stem cells of adult patients with atopic dermatitis: At the origin of the problem. Br. J. Dermatol..

[60] Ahn H., Lee S.Y., Jung W.J., Pi J., Lee K.H. (2021). Psoriasis treatment using minimally manipulated umbilical cord-derived mesenchymal stem cells: A case report. World J. Clin. Cases.

[61] Weise G., Lorenz M., Pösel C., Maria Riegelsberger U., Störbeck V., Kamprad M., Kranz A., Wagner D.C., Boltze J. (2014). Transplantation of cryopreserved human umbilical cord blood mononuclear cells does not induce sustained recovery after experimental stroke in spontaneously hypertensive rats. J. Cereb. Blood Flow Metab..

[62] Antebi B., Asher A.M., Rodriguez L.A., Moore R.K., Mohammadipoor A., Cancio L.C. (2019). Cryopreserved mesenchymal stem cells regain functional potency following a 24-h acclimation period. J. Transl. Med..

[63] Lukomska B., Stanaszek L., Zuba-Surma E., Legosz P., Sarzynska S., Drela K. (2019). Challenges and Controversies in Human Mesenchymal Stem Cell Therapy. Stem Cells Int..

[64] Marks P.W. (2020). Clear Evidence of Safety and Efficacy Is Needed for Stromal Vascular Fraction Products: Commentary on “Arguments for a Different Regulatory Categorization and Framework for Stromal Vascular Fraction”. Stem Cells Dev..

